# The impact of concurrency conflicts on optimal location of air ambulance bases in Norway

**DOI:** 10.1186/s13049-025-01324-3

**Published:** 2025-04-04

**Authors:** Jo Røislien, Pieter L. van den Berg, J. Theresia van Essen, Oddvar Uleberg, Caroline Jagtenberg

**Affiliations:** 1 Department of Research, The Norwegian Air Ambulance Foundation, Oslo, Norway; 2 Faculty of Health Sciences, University of Stavanger, Stavanger, Norway; 3 Department of Emergency Medicine and Pre-Hospital Services, St. Olav’s University Hospital, Trondheim, Norway; 4 Department of Circulation and Medical Imaging, Norwegian University of Science and Technology, Trondheim, Norway; 5 Delft Institute of Applied Mathematics, Delft University of Technology, Delft, the Netherlands; 6 Department of Technology and Operations Management, Rotterdam School of Management, Erasmus University, Rotterdam, the Netherlands; 7 Department of Operations Analytics, School of Business and Economics, Vrije Universiteit Amsterdam, Amsterdam, the Netherlands

**Keywords:** HEMS, Air ambulance, Facility location problem, MCLP, MEXCLP, Incidents, Coverage, Busy fraction, Concurrency conflicts

## Abstract

**Background:**

Helicopter emergency medical services (HEMS) are important in many health care systems. In order to best utilize this expensive healthcare service, the location of HEMS bases is key. Concurrency conflicts is a prominent deviation for not completing missions, yet is often overlooked in mathematical modelling. The aim of the present study was to calculate optimal air ambulance base locations when accounting for the potential unavailability of helicopters due to concurrency conflicts.

**Methods:**

We used incident data for Norway from 2015. Optimal helicopter base locations were estimated using the Maximum Expected Covering Location Problem (MEXCLP) optimization model, allowing for estimation of the impact of concurrency conflicts by introducing a busy fraction parameter in the model. We explored busy fractions of 0, 0.10, 0.20 and 0.30, representing helicopters on the HEMS bases being busy 0, 10, 20 and 30% of the time, respectively. Both greenfield scenarios and simulations conditioned on the existing base structure were explored.

**Results:**

The 428 municipalities had a median (5–95 percentile) of 10 (2–38) incidents. Assuming a helicopter is always available, the existing bases cover an estimated 73.6% of the incidents within 30 min. Increasing the busy fraction in the calculations resulted in a significant decrease in estimated coverage. Re-arranging the currently available 14 helicopters in a greenfield analysis increases coverage to 91.9%. Increasing the busy fraction in the models, the mathematically optimal solutions put increasingly more emphasis on the more densely populated greater Oslo area, removing helicopters from northern Norway and the coastal areas, where population is more spread.

**Conclusion:**

The busy fraction significantly impacts the optimal location of air ambulance bases, with higher busy fractions resulting in more helicopters being placed in the more densely populated areas where demand is higher. However, the actual busy fractions reported in the Norwegian HEMS system seem to be of a magnitude small enough to have little impact on the optimal location of HEMS bases and helicopters. To determine the impact of adjusting for non-homogeneous busy fractions across the country more refined busy fraction models are needed.

## Background

Helicopter emergency medical services (HEMS) are an important part of the health care system in many developed countries [1, 2], and is expanding throughout the world. The service helps provide access to remote areas, brings advanced treatment options and decision-making competence to the scene of the emergency, and reduces transport times [3–5].

Norway’s population is heterogeneously spread throughout the country, ranging from densely populated urban areas, to large, sparsely populated regions. A paramount principle in Norwegian health legislation is that all citizens should have equal access to publicly funded health care regardless of their residential pattern [6]. HEMS is considered essential in order to achieve this goal, and the objective of the Norwegian air ambulance service is to provide advanced emergency medicine to critically ill or severely injured patients. The service is a public nationwide anesthesiologist manned air ambulance service, operating 24/7/365.

HEMS is an expensive healthcare service. In order to best utilize the service’s resource, ensuring coverage of the largest part of the population, in the shortest amount of time, so that incidents can be served as quickly as possible, the location of the HEMS bases is key. Currently, there are 14 helicopter ambulances spread out over 13 bases in Norway, established gradually through historical local engagement from the late 1970s [7]. Over the last years, however, the optimal location of HEMS bases has been explored mathematically in several publications [8–13]. Central in many of these papers is the Maximum Coverage Location Problem (MCLP) [14], a mathematical model that can be used for determining the highest possible coverage for a pre-specified number of bases.

While the MCLP is generally regarded as a robust method for locating emergency vehicles, the MCLP model assumes that an emergency medical services (EMS) vehicle is always available at the base whenever needed [15]. In reality, this is often not the case. A large percentage of HEMS flights experience deviations from standard operational implementation, including cancellations or aborted missions due to weather, technical issues, duty time limitations, or no medical need [16]. Among the more prominent deviations are concurrency conflicts, constituting a well-known and regular phenomenon for those who work within EMS services [16].

The Maximum Expected Covering Location Problem (MEXCLP) [17] models EMS vehicle coverage more realistically by taking the potential unavailability of vehicles into account by including a busy fraction parameter. The more refined MEXCLP model thus calculates not just the mathematically optimal location of each base but also the number of vehicles serving from each base [18]. For EMS, an analysis using MEXCLP for the optimal location of ground vehicles and bases was performed for a localized region of Norway [18]. To the best of our knowledge, no similar analysis has been explored for HEMS. While more advanced models exist, none of these consider a scenario allowing for busy fractions to differ per vehicle and depend on the availability of other vehicles. Using MEXCLP is thus a valuable first step for evaluating how including busy fractions might impact results in terms of optimizing HEMS base locations.

The aim of the present analysis is to explore the optimal location of HEMS bases in Norway when taking concurrency conflicts into account. By varying the model’s busy fraction in a series of simulation studies, both using the existing base structure as a starting point, and in greenfield analyses assuming a clean slate, we explore the sensitivity to differences in unavailability of helicopters on potential geographical re-location of bases.

## Materials and methods

### Data material

**Fig. 1:**
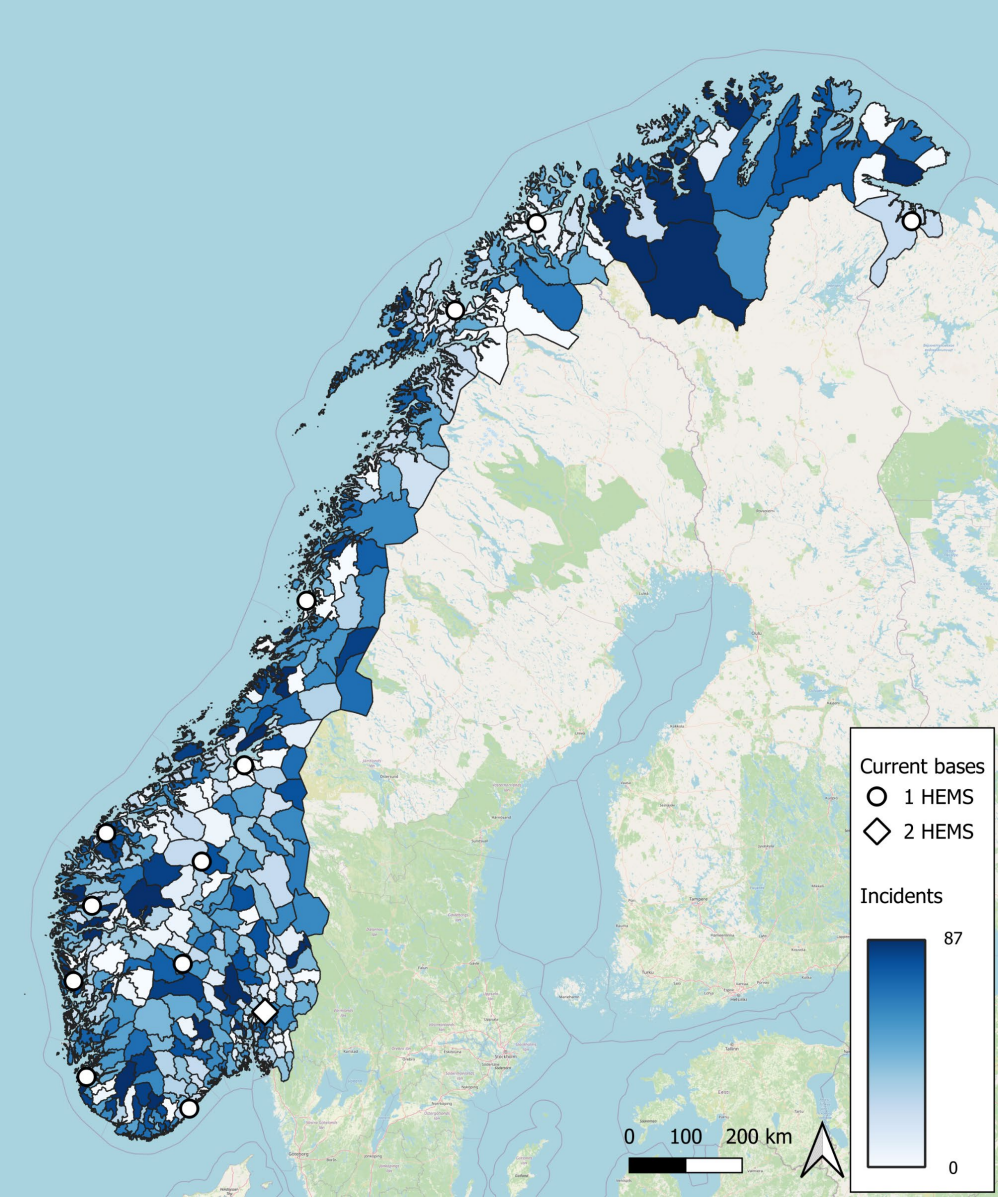
The 428 municipalities in Norway in 2015, with colors indicating the total number of incidents that year, along with the current 13 HEMS bases

Norway is a long and narrow country located at the far North of Europe, stretching 1790 km from north to south, covering an area of 323,802 km^2^. The country has a mixed rural and urban population with county population density ranging from 1129.5 inh/km^2^ in Oslo to 1.5 inh/km^2^ in the northernmost county of Finnmark. January 1st 2015, the population in Norway was 5.2 million [10], with around one third located in the vicinity of the capital, Oslo. Servicing this population, there are currently 14 HEMS units positioned on 13 bases spread across the country (Fig. 1).

Despite large geographical distances and substantial uninhabited areas, government requirements state that 90% of the population should be reached by a physician manned ambulance service within 45 min [19]. The existing base structure already covers 96.9% of the population within 45 min and 84.7% within 30 min [10]. Given the increased focus on lowering response times, we used a threshold of 30 min in the current calculations.

In 2015, Norway consisted of 428 municipalities. Population density data has been shown to be poorly correlated with actual incidences [10] and we thus used incident data rather than population density data in the present work. Aggregated yearly municipality incident data for primary acute missions are available from the National Air Ambulance Services upon request. In 2015, the number of incidents per municipality had a median (5–95 percentile) of 10 [2–38], with about 70% of the missions being medical, and 30% trauma [20]. Called off cases are not included in the analyses.

The average pre-flight preparation time for Norwegian HEMS operations is 5.5 min [6] and this number was used in the mathematical calculations. Helicopter ground speed depends on weather conditions. In the mathematical models, we used 220 km/h, as an overall average number, taking into account the different helicopter types and the helicopter speeds used during each mission (i.e. take-off, cruise phase, and landing phase including identification of suitable landing sites).

There are multiple reasons why dispatched HEMS missions are not completed. All HEMS dispatches in Norway are electronically registered. The underlaying reason as to why a mission was either cancelled or aborted, both referred to as “deviations”, is also registered as one of seven options: “No medical need”, “Patient not transport capable”, “Dead on scene”, “Technical issue”, “Weather”, “Duty time limitation” or “Concurrency conflict”. Numbers on deviations in the air ambulance service are published in the annual reports from the National Air Ambulance Service [21]. The number of concurrency conflicts for the years 2004 through 2014, along with total number of missions, allows for the calculation of the busy fraction for each of the 12 bases during the 11-year period. These numbers were then used to guide the input parameters to the mathematical models and ensure that our experiments represent realistic scenarios.

### Mathematical modelling

In the present analyses, all 428 municipalities were used as both demand locations and potential base locations, more specifically the population-weighted centroids within each municipality. The travel times, including the 5.5-min fixed pre-flight preparation time, from all potential base locations to all demand locations were then calculated, that is, *from* all municipalities *to* all municipalities, as input to the mathematical optimization.

Optimal base locations were first determined by modelling the problem as a Maximal Covering Location Problem (MCLP) [14]. The MCLP model maximizes the number of demand locations covered by at least one helicopter, weighted by the number of incidents in each demand location. In the MCLP model, it is assumed that an emergency vehicle is always available at a base whenever needed. As such, the model represents a best-case scenario, as demand is modeled as completely covered if the demand point, i.e. the municipality, is within the reach of a facility location, i.e. a HEMS base.

We then applied the more refined MEXCLP model [17, 18], allowing for the situation that a helicopter can be temporarily busy. This is done by introducing a busy fraction parameter into the equations, denoting the fraction of time a helicopter is busy. The MEXCLP can thus model the situation that, when a demand point is potentially covered by *two* helicopters, of which one is currently busy, the second is able to service this demand. Moreover, the model also allows putting multiple helicopters at the same base location. The MEXCLP model assumes that helicopters are independent and that the busy fraction for all helicopters and air ambulance bases is the same [15]. With a busy fraction of zero, the MEXCLP model is equivalent to the simpler MCLP model.

Using 2015 incident data, we calculated optimal base locations assuming no bases existed, so-called greenfield analysis, inserting both 13, 14 and 15 helicopters, to explore potential loss or gain in varying the number of helicopters. In all three cases, we further explored how the optimal helicopter locations changed, as well as how the coverage would change with increasing busy fractions of 0, 0.1, 0.2 and 0.3, representing that helicopters at the HEMS bases on average are busy 0, 10, 20 and 30% of the time.

All models were implemented in Python 3.9.5, using the PuLP 2.6.0 package [22] and its default solver CBC [23].

## Results

**Fig. 2:**
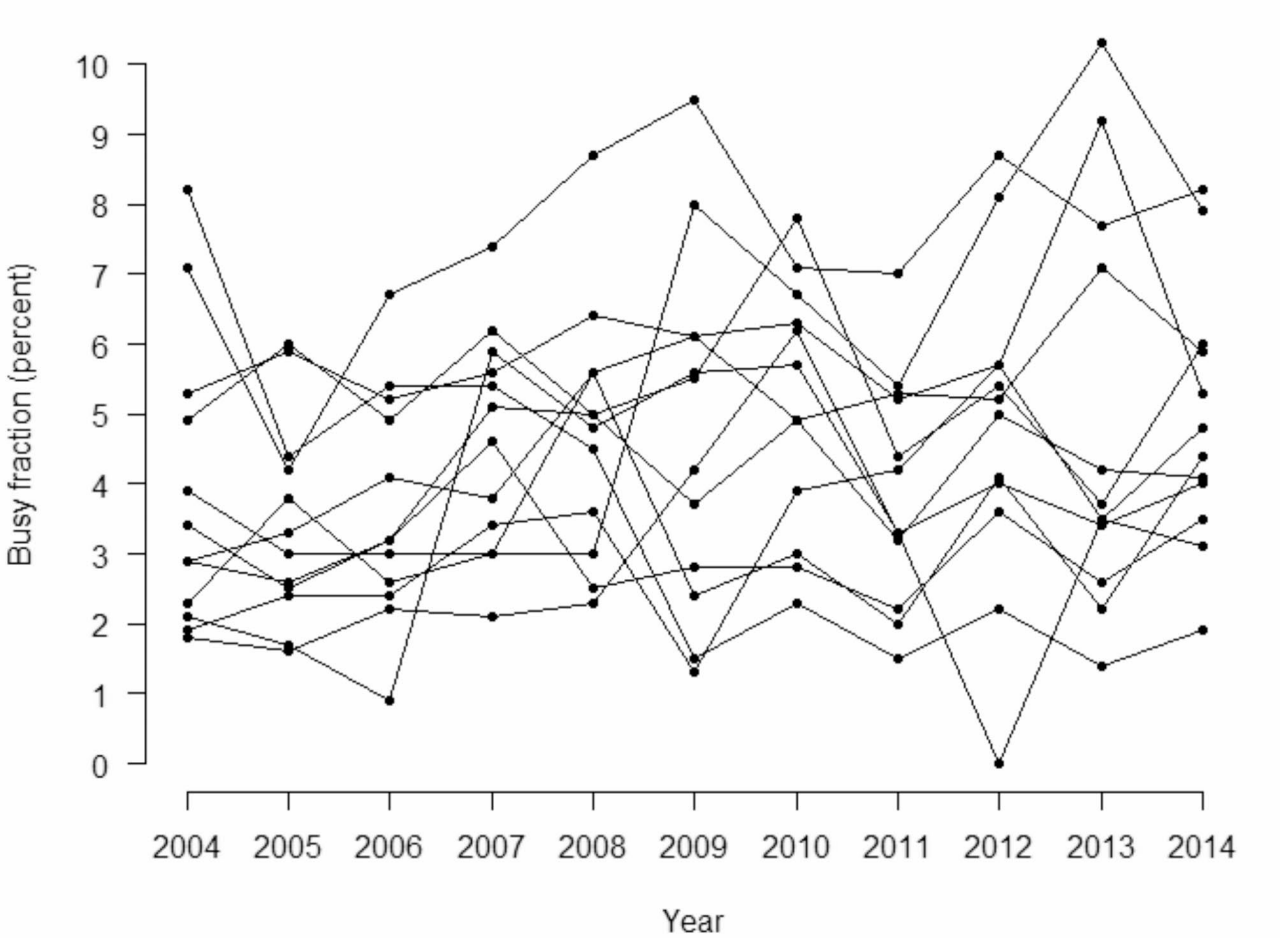
Busy fractions for the 12 Norwegian HEMS bases 2004–2014, demonstrating variation both between and within bases from year to year

In 2015, for 9 out of the then 11 bases, concurrency conflicts were the third most prominent deviation for not completing missions, beaten only by “Weather” and “No medical need” [20]. Busy fractions for individual HEMS bases for the 11-year period of 2004–2014 demonstrate that busy fractions vary between bases, as well as within bases, from year to year (Fig. 2). Overall, busy fractions for all bases taken together range from 0 to 10.3%, with a grand mean of 4.4%.

The existing base structure (Fig. 1) has a coverage of 73.6%, assuming a helicopter is always available at the nearest base (Table 1). For busy fractions 0.1, 0.2 and 0.3, the coverage drops to 68.2%, 62.4% and 56.1%, respectively.

**Table 1:** Percent coverage for different models and busy fractions, both for the existing base structure and greenfield analysis with different number of bases

		MCLP (single coverage)	MEXCLP (backup coverage from multiple bases and helicopters)		
		Busy fraction			
	Number of helicopters	0.0	0.1	0.2	0.3
Existing base structure	14	73.6%	68.2%	62.4%	56.1%
Greenfield analysis	13	89.8%	81.6%	74.2%	67.0%
	14	91.9%	84.1%	76.6%	69.2%
	15	94.1%	86.2%	78.8%	71.3%

**Fig. 3:**
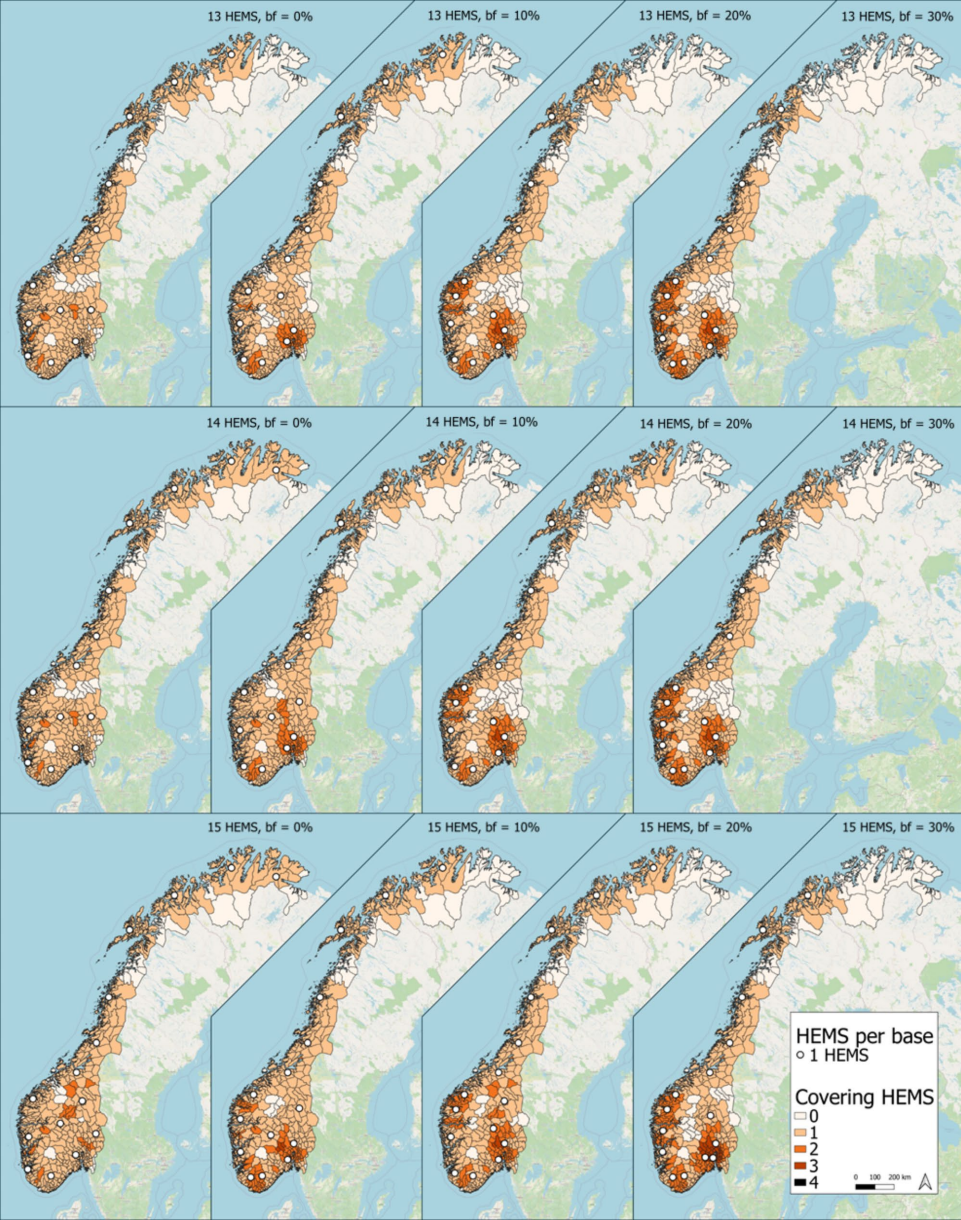
Optimal location of 13, 14 or 15 bases (rows), using a 30-minute threshold and busy fractions (bf) of 0, 10, 20 and 30% (columns)

In a greenfield analysis, solving a MCLP, i.e., a MEXCLP with busy fraction 0%, with 14 air ambulances gives a coverage of 91.9% (Table 1), as compared to 73.6% for the existing base structure. The optimal base structure is markedly different to the existing one (Fig. 3).

Increasing the busy fraction decreases the coverage (Table 1), and results in a reorganization of the bases. Increasing to a busy fraction of 10% increases the need for helicopters in the densely population Oslo region, at the expense of one of the helicopters in the more sparsely populated Northern Norway (Fig. 3). Increasing to 20% further amplifies this effect, now at the expense of one of the helicopters at the coast in middle Norway. Increasing to 30% removes yet another helicopter in Northern Norway and places it in the larger Oslo vicinity.

Reducing the number of helicopters from 14 to 13, in a mathematically optimal situation, does not lead to a large decrease in coverage. This even holds with increasing busy fractions. The incremental gain of adding a 15th helicopter in a mathematical greenfield analysis is also slim (Table 1).

## Discussion

The results from the mathematical experiments presented in this paper indicate that taking into account that helicopters might not always be available whenever needed, will impact the optimal location of HEMS bases.

In the present analyses, we have applied a more advanced mathematical model than the MCLP often used in this type of studies of HEMS base locations, by including a busy fraction parameter. With increasing busy fractions, the optimal solution of the model allocates more of the resources, and thus HEMS bases, closer to areas where demand is clustered. In particular, we see increasingly more HEMS bases re-located into the larger Oslo area (i.e., the higher population density area), at the cost of reducing the number of bases in the northern part of the country and along the coast where the population is more scattered.

The fact that a helicopter can be temporarily unavailable, modelled by the busy fraction, highlights the importance of back-up capacity. That is, demand points, i.e., municipalities, with high demand should be covered by multiple helicopters. The MEXCLP results demonstrate this: a clustering of helicopters in the Greater Oslo region yields a set of demand points that is covered by up to four helicopters. Note that this does not necessarily indicate that the model assigns the Greater Oslo region a 4th helicopter over giving people in the North a 1st helicopter: the few municipalities covered four times is most likely a side effect of the model’s intention to cover other municipalities three times.

In the MEXCLP model used in the present analyses, the busy fraction is assumed to be similar for all bases in the model. Empirical data from concurrency conflicts for individual bases demonstrates that this is not the case (Fig. 2). Rather, the busy fraction varies across the different bases throughout the country. More sophisticated models should thus be pursued, to explore the effect this might have on determining optimal HEMS base locations. It is however worth noting that the busy fractions observed in the Norwegian HEMS system is generally below 10% (Fig. 2), which is lower than most busy fractions explored in this computational experiment, having marginal effect on the optimal location of HEMS bases and helicopters (Fig. 3). This lends weight to previous studies on the same topic, using the simpler MCLP model.

The MCLP and MEXCLP are well known and flexible mathematical models that allow for different levels of modelling complexity, depending on the question at hand. Ahmed et al. [24] present an iterative modelling approach combing both the MCLP and MEXCLP model with a variety of healthcare and transportation data. An underlying assumption of the MEXCLP model however is that bases and their assigned helicopters become busy independently of one another. In reality, however, when many helicopters at bases close to one another are busy, the remaining idle ones have a higher-than-average chance of also becoming busy: when the next call comes in, it is more likely that *they* will now be the nearest available helicopter. That is, busyness is not entirely independent between bases, particularly for nearby helicopters. So, while the MEXCLP is more sophisticated than the MCLP, it too is a simplification of reality. The Adjusted MEXCLP (AMEXCLP) [25] models this dependence between bases better, by embedding correction factors into the MEXCLP’s mathematical formulations. This was originally defined based on a hypercube queuing model [26, 27]. Neither of these models do however overcome the issue that each helicopter in the system is assigned the same busy fraction. Compared to the MEXCLP model, these models are also considerably more complicated to grasp and more complex to implement. Runtimes are also a concern [28].

Since HEMS represents a highly specialized and costly service, patient benefit must continuously be balanced against costs, operational risk and unnecessary flights [5, 29]. Proper use of HEMS has become a progressively more discussed topic in prehospital emergency research [30, 31]. Ideally, the rationale behind dispatching HEMS to a patient should involve either enhancing on-site expertise by providing advanced interventions that are not available through other EMS or providing essential logistical support. Ulvin et al. [32] recently showed that after introducing a dedicated HEMS coordinator and revised dispatch criteria, a significantly higher mean severity score (i.e. National advisory committee for aeronautics - NACA) [33] and a higher proportion of patients with severe illness or injury (i.e. NACA 4–7) were found in the post intervention group [29]. Better precision in use, results in a lower busy fraction and increased coverage. Managers of HEMS should thus explore how the busy fraction could be reduced, be it by e.g. shortening handover time or reducing the number of unnecessary flights by improved triage.

In the MEXCLP model used in the present study, the demand is assumed to be static. In reality, demand will likely fluctuate with time, and busy fractions will accordingly differ temporally. In Norway, weather varies strongly throughout the year, affecting where Norwegians spend their time: in winter, many Norwegians find their way to the snow-covered mountains, while during summer, they spend time by the coast. Seasonal and weather-related variations in the number of trauma admissions has been found in several studies [34–36]. Seasonal effects on busy fractions might thus be significant– maybe even on shorter time intervals as well: In temporal modelling of trauma admissions, a weekly cycle effect was found to be statistically significant in all fitted statistical models [36]. In a study of EMS ground vehicles at a Norwegian healthcare trust, both daily and weekly temporal effects were significant factors for organizing the service efficiently [18]. Mathematical models that include temporal behavior of the busy fraction exist: they effectively split time in a number of predefined disjoint intervals and either optimize for each interval independently [37] or penalize the number of relocations between intervals [38]. Exploring this does however take more temporally refined data than yearly summaries.

When optimizing for efficiency, serving the largest possible portion of incidents within a pre-defined time frame is an important goal of the HEMS. As for all healthcare activity, resources are limited, and must be utilized in the best way possible. The present analysis indicates how optimal resource distribution changes as the busy fraction at individual bases increases. Reducing the busy fraction of helicopters will thus have a positive impact. Notably, however, this study demonstrates that busy fractions reported in the Norwegian HEMS system are generally of a magnitude small enough to have little impact on the optimal solution of HEMS bases and helicopters. The impact of adjusting for non-homogeneous busy fractions across the country is thus hard to quantify. More detailed studies into busy fractions and their accompanying mathematical models are needed before concluding.

## Conclusion

Concurrency conflicts are among the most prominent deviations for not completing HEMS missions. In the present study concurrency conflicts were accounted for by introducing a busy fraction in the mathematical modelling, and results indicate that the busy fraction significantly impacts the optimal location of air ambulance bases, with higher busy fractions resulting in more helicopters being placed in the more densely populated areas where demand is higher. The actual busy fractions reported in the Norwegian HEMS system do however appear to be of a magnitude small enough to have little impact on the optimal solution of HEMS bases and helicopters. To determine the impact of adjusting for non-homogeneous busy fractions across the country more refined busy fraction models are needed.

## Data Availability

Incident data is available upon reasonable request.

## References

[1] Garner AA. The role of physician staffing of helicopter emergency medical services in prehospital trauma response. Emerg Med Australas. 2004;16(4):318–23. 15283719 10.1111/j.1742-6723.2004.00636.x

[2] Taylor CB, Stevenson M, Jan S, Liu B, Tall G, Middleton PM, et al. An investigation into the cost, coverage and activities of Helicopter Emergency Medical Services in the state of New South Wales, Australia. Injury. 2011;42(10):1088–94. 21459379 10.1016/j.injury.2011.02.013

[3] Krüger AJ, Skogvoll E, Castrén M, Kurola J, Lossius HM. Scandinavian pre-hospital physician-manned Emergency Medical services–same concept across borders? Resuscitation. 2010;81(4):427–33. 20122784 10.1016/j.resuscitation.2009.12.019

[4] Hesselfeldt R, Steinmetz J, Jans H, Jacobsson ML, Andersen DL, Buggeskov K, et al. Impact of a physician-staffed helicopter on a regional trauma system: a prospective, controlled, observational study. Acta Anaesthesiol Scand. 2013;57(5):660–8. 23289798 10.1111/aas.12052 PMC3652037

[5] Floccare DJ, Stuhlmiller DF, Braithwaite SA, Thomas SH, Madden JF, Hankins DG, et al. Appropriate and safe utilization of helicopter emergency medical services: a joint position statement with resource document. Prehosp Emerg Care. 2013;17(4):521–5. 23834231 10.3109/10903127.2013.804139

[6] Zakariassen E, Uleberg O, Røislien J. Helicopter emergency medical services response times in Norway: do they matter? Air Med J. 2015;34(2):98–103. 25733116 10.1016/j.amj.2014.11.003

[7] Steen PA, Heggestad T. Luftambulansetjenesten i Norge. Nord Med. 1994;109(10):263–4. 7937020

[8] Jansen JO, Morrison JJ, Wang H, He S, Lawrenson R, Hutchison JD, et al. Access to specialist care: optimizing the geographic configuration of trauma systems. J Trauma Acute Care Surg. 2015;79(5):756–65. 26335775 10.1097/TA.0000000000000827 PMC4623849

[9] Røislien J, van den Berg PL, Lindner T, Zakariassen E, Aardal K, van Essen JT. Exploring optimal air ambulance base locations in Norway using advanced mathematical modelling. Inj Prev. 2017;23(1):10–5. 27325670 10.1136/injuryprev-2016-041973 PMC5293838

[10] Røislien J, van den Berg PL, Lindner T, Zakariassen E, Uleberg O, Aardal K, et al. Comparing population and incident data for optimal air ambulance base locations in Norway. Scand J Trauma Resusc Emerg Med. 2018;26(1):42. 29793526 10.1186/s13049-018-0511-4 PMC5968535

[11] Jagtenberg CJ, Vollebergh MAJ, Uleberg O, Røislien J. Introducing fairness in Norwegian air ambulance base location planning. Scand J Trauma Resusc Emerg Med. 2021;29(1):50. 33743747 10.1186/s13049-021-00842-0 PMC7980553

[12] Jagtenberg CJ, Uleberg O, Waaler Bjørnelv GM, Røislien J. Utopia for Norwegian helicopter emergency medical services: estimating the number of bases needed to radically bring down response times, and lives needed to be saved for cost effectiveness. PLoS ONE. 2023;18(3):e0281706. 36996062 10.1371/journal.pone.0281706 PMC10062538

[13] Heggestad T, Børsheim KY. Accessibility and distribution of the Norwegian National Air Emergency Service: 1988–1998. Air Med J. 2002;21(3):39–45. 11994734 10.1067/mmj.2202.124220

[14] Church R, ReVelle C, editors. The maximal covering location problem. Papers of the regional science association. Berlin/Heidelberg: Springer-; 1974.

[15] Li X, Zhao Z, Zhu X, Wyatt T. Covering models and optimization techniques for emergency response facility location and planning: a review. Math Methods Oper Res. 2011;74(3):281–310.

[16] Østerås Ø, Brattebø G, Heltne JK. Helicopter-based emergency medical services for a sparsely populated region: a study of 42,500 dispatches. Acta Anaesthesiol Scand. 2016;60(5):659–67. 26810562 10.1111/aas.12673 PMC5064740

[17] Daskin MS. A Maximum Expected Covering Location Model: Formulation, Properties and Heuristic Solution. Transport Sci. 1983;17(1):48–70.

[18] van den Berg PL, Fiskerstrand P, Aardal K, Einerkjær J, Thoresen T, Røislien J. Improving ambulance coverage in a mixed urban-rural region in Norway using mathematical modeling. PLoS ONE. 2019;14(4):e0215385. 30978264 10.1371/journal.pone.0215385 PMC6461285

[19] services. NGMohac. White Paper Report No. 43 (1999–2000): about emergency medical preparedness. 2000.

[20] Foundation TNAA. Capacity and base structure– A report on the Norwegian Air Ambulance Service 1988–2011. 2013 September.

[21] Aktivitetsrapport for. luftambulansetjenesten 2022. 2022.

[22] Mitchell S, O’Sullivan M, Dunning I, editors. PuLP: A Linear Programming Toolkit for Python2011.

[23] John Forrest TR, Stefan Vigerske, Haroldo Gambini Santos, John Forrest, Lou Hafer,Bjarni Kristjansson, jpfasano, EdwinStraver, Jan-Willem, Miles Lubin, rlougee, a-andre,jpgoncal1, Samuel Brito, h-i-gassmann, Cristina, Matthew Saltzman, tosttost,… to-st.coin-or/Cbc. releases/2.10.12 ed. Zenodo2024.

[24] Ahmed S, Lieberthal RD, Hechtman DM, Rayson LA, Amirault DR, Haas S. Framework for Optimizing Air Ambulance Locations. AMIA Jt Summits Transl Sci Proc. 2022;2022:102– 11. PMC9285155 35854752

[25] Batta R, Dolan JM, Krishnamurthy NN. The maximal Expected Covering Location Problem: Revisited. Transport Sci. 1989;23(4):277–87.

[26] Larson RC. A hypercube queuing model for facility location and redistricting in urban emergency services. Comput Oper Res. 1974;1(1):67–95.

[27] Larson RC. A Hypercube Queueing Model for Facility Location and Redistricting in Urban Emergency services. Santa Monica, CA: RAND Corporation; 1973.

[28] Saydam C, Aytuğ H. Accurate estimation of expected coverage: revisited. Socio-Economic Plann Sci. 2003;37(1):69–80.

[29] Ulvin OE, Skjærseth E, Haugland H, Thorsen K, Nordseth T, Orre MF, et al. The introduction of a regional Norwegian HEMS coordinator: an assessment of the effects on response times, geographical service areas and severity scores. BMC Health Serv Res. 2022;22(1):1020. 35948977 10.1186/s12913-022-08337-z PMC9365225

[30] Adcock AK, Minardi J, Findley S, Daniels D, Large M, Power M. Value Utilization of Emergency Medical Services Air Transport in Acute ischemic stroke. J Emerg Med. 2020;59(5):687–92. 33011044 10.1016/j.jemermed.2020.08.005 PMC8006070

[31] Vercruysse GA, Friese RS, Khalil M, Ibrahim-Zada I, Zangbar B, Hashmi A, et al. Overuse of helicopter transport in the minimally injured: a health care system problem that should be corrected. J Trauma Acute Care Surg. 2015;78(3):510–5. 25710420 10.1097/TA.0000000000000553

[32] Ulvin OE, Skjærseth E, Krüger AJ, Thorsen K, Nordseth T, Haugland H. Can video communication in the emergency medical communication centre improve dispatch precision? A before-after study in Norwegian helicopter emergency medical services. BMJ Open. 2023;13(10):e077395. 37899141 10.1136/bmjopen-2023-077395 PMC10618992

[33] Weiss M, Bernoulli L, Zollinger A. [The NACA scale. Construct and predictive validity of the NACA scale for prehospital severity rating in trauma patients]. Anaesthesist. 2001;50(3):150–4. 11315486 10.1007/s001010170030

[34] Pape-Köhler CI, Simanski C, Nienaber U, Lefering R. External factors and the incidence of severe trauma: time, date, season and moon. Injury. 2014;45(Suppl 3):S93–9. 25284243 10.1016/j.injury.2014.08.027

[35] Bhattacharyya T, Millham FH. Relationship between Weather and Seasonal factors and trauma admission volume at a level I trauma Center. J Trauma Acute Care Surg. 2001;51(1). 10.1097/00005373-200107000-00019 11468478

[36] Røislien J, Søvik S, Eken T. Seasonality in trauma admissions - are daylight and weather variables better predictors than general cyclic effects? PLoS ONE. 2018;13(2):e0192568. 29425210 10.1371/journal.pone.0192568 PMC5806884

[37] Repede JF, Bernardo JJ. Developing and validating a decision support system for locating emergency medical vehicles in Louisville, Kentucky. Eur J Oper Res. 1994;75(3):567–81.

[38] van den Berg PL, Aardal K. Time-dependent MEXCLP with start-up and relocation cost. Eur J Oper Res. 2015;242(2):383–9.

